# MicroRNA-212/ABCG2-axis contributes to development of imatinib-resistance in leukemic cells

**DOI:** 10.18632/oncotarget.21272

**Published:** 2017-09-26

**Authors:** Meike Kaehler, Johanna Ruemenapp, Daniel Gonnermann, Inga Nagel, Oliver Bruhn, Sierk Haenisch, Ole Ammerpohl, Daniela Wesch, Ingolf Cascorbi, Henrike Bruckmueller

**Affiliations:** ^1^ Institute of Experimental and Clinical Pharmacology, University Hospital Schleswig-Holstein, Campus Kiel, Kiel, Germany; ^2^ Institute of Immunology, University Hospital Schleswig-Holstein, Campus Kiel, Kiel, Germany; ^3^ Institute of Human Genetics, University Hospital Schleswig-Holstein, Campus Kiel, Kiel, Germany

**Keywords:** drug resistance, drug transporters, ABCG2, miR-212, methylation

## Abstract

BCR-ABL-independent resistance against tyrosine kinase inhibitor is an emerging problem in therapy of chronic myeloid leukemia. Such drug resistance can be linked to dysregulation of ATP-binding cassette (ABC)-transporters leading to increased tyrosine kinase inhibitor efflux, potentially caused by changes in microRNA expression or DNA-methylation. In an *in vitro*-imatinib-resistance model using K-562 cells, microRNA-212 was found to be dysregulated and inversely correlated to ABC-transporter ABCG2 expression, targeting its 3′-UTR. However, the functional impact on drug sensitivity remained unknown. Therefore, we performed transfection experiments using microRNA-mimics and –inhibitors and investigated their effect on imatinib-susceptibility in sensitive and resistant leukemic cell lines. Under imatinib-treatment, miR-212 inhibition led to enhanced cell viability (*p* = 0.01), reduced apoptosis (*p* = 0.01) and cytotoxicity (*p* = 0.03). These effects were limited to treatment-naïve cells and were not observed in cells, which were resistant to various imatinib-concentrations (0.1 μM to 2 μM). Further analysis in treatment-naïve cells revealed that miR-212 inhibition resulted in ABCG2 upregulation and increased ABCG2-dependent efflux. Furthermore, we observed *miR-212* promoter hypermethylation in 0.5 and 2 μM IM-resistant sublines, whereas *ABCG2* methylation status was not altered. Taken together, the miR-212/ABCG2-axis influences imatinib-susceptibility contributing to development of imatinib-resistance. Our data reveal new insights into mechanisms initiating imatinib-resistance in leukemic cells.

## INTRODUCTION

Pharmacotherapy with tyrosine kinase inhibitors (TKI) is currently the first-line treatment in chronic myeloid leukemia (CML). Despite the tremendous success of TKIs such as imatinib mesylate (IM) or nilotinib the development of drug-resistance during the course of treatment is still a major problem [1, 2]. Resistances against IM occur due to different BCR-ABL-dependent mechanisms i.e. mutations in BCR-ABL kinase domain or overexpression of the *BCR-ABL* fusion gene. Furthermore, they can arise BCR-ABL-independently [2], i.e. due to overexpression of drug efflux transporters, such as ABCB1 (P-glycoprotein, P-gp) and ABCG2 (breast cancer resistance protein, BCRP), known for their crucial role in pharmacoresistance [3–6]. In IM-resistance, however, the contribution of these two transporters is controversially discussed, as there are reports on upregulation of ABCB1 [7, 8] or ABCG2 [9–11]. There are several hints that the expression of both drug transporters is dynamic during the development of IM-resistance and might change during early and late IM-resistance [12]. Nevertheless, the regulatory processes and clinical relevance is still not fully understood. In a previous study, we demonstrated a dynamic regulation of ABCG2 during the development of IM-resistance *in vitro*, showing peak expression at low IM doses (0.1 to 0.5 μM) and low expression at high IM doses (2 μM) [11].

Aside ABC-transporter induction through nuclear receptors such as the pregnane X-receptor (PXR) and the constitutive androstane receptor (CAR), there is increasing evidence that drug transporter expression is regulated by microRNAs [13–17]. Those 17–24 nt long non-coding RNAs are involved in the posttranscriptional regulation of their target mRNAs. In the last years, the relevance of microRNAs in diseases became more obvious, being dysregulated in a variety of cancers [18, 19]. MiR-212 is one microRNA, which is often dysregulated in cancer and might function as a tumor suppressor. It is described to influence tumor necrosis factor-related apoptosis-inducing ligand (TRAIL)-sensitivity in non-small lung cancer and B-cell receptor expression in CML [20, 21]. Furthermore, miR-212 can be considered as a prognostic marker in acute myeloid leukemia (AML) [22]. In our previous study, we observed an inverse correlation of miR-212 and miR-328 to ABCG2 during the development of IM-resistance [11]. In addition, these data revealed that the 3′-UTR of *ABCG2* is a direct target of miR-212, but not of miR-328. However, it remained unknown whether changes in miR-212 expression contributed to alterations in drug transport-related IM-sensitivity.

In addition to posttranscriptional regulation, there is increasing evidence that expression of *ABCB1* and *ABCG2* is *-* at least partially - regulated by promoter methylation. In several cancer entities, aberrant *ABCB1* or *ABCG2* methylation was shown, i.e. in leukemia or solid tumors [23–25] and was also observed in drug-resistance [26, 27]. In CML patients, aberrant DNA methylation of various genes could be associated to disease progression and potentially, IM-resistance [28]. However, it remained unknown, if methylation patterns of these genes were changed during the development of IM-resistance. There are several hints that miR-212 expression is regulated by methylation and can be deregulated in cancer. In solid tumors, such as lung and gastric cancer, methylation was shown to function as an epigenetic modulator of miR-212 expression [29, 30]. Nevertheless, the influence of methylation on *miR-212* in CML remains unknown.

In this study, we investigated the effects of miR-212 on IM-susceptibility during the development of IM-resistance. For this purpose, we analyzed the impact of miR-212 on cell survival, viability and apoptosis in treatment-naïve and IM-resistant K-562 cells using loss and gain of function experiments. Furthermore, we examined the link between miR-212 and ABCG2 expression and function by flow cytometry and a transport assay. To elucidate how promoter methylation is altered during the development of IM-resistance, we performed *in vitro*-analyses of various CpG islands of *ABCB1*, *ABCG2* and *miR-212*.

## RESULTS

### miR-212 and ABCG2 expression during the development of imatinib-resistance

**Figure 1:**
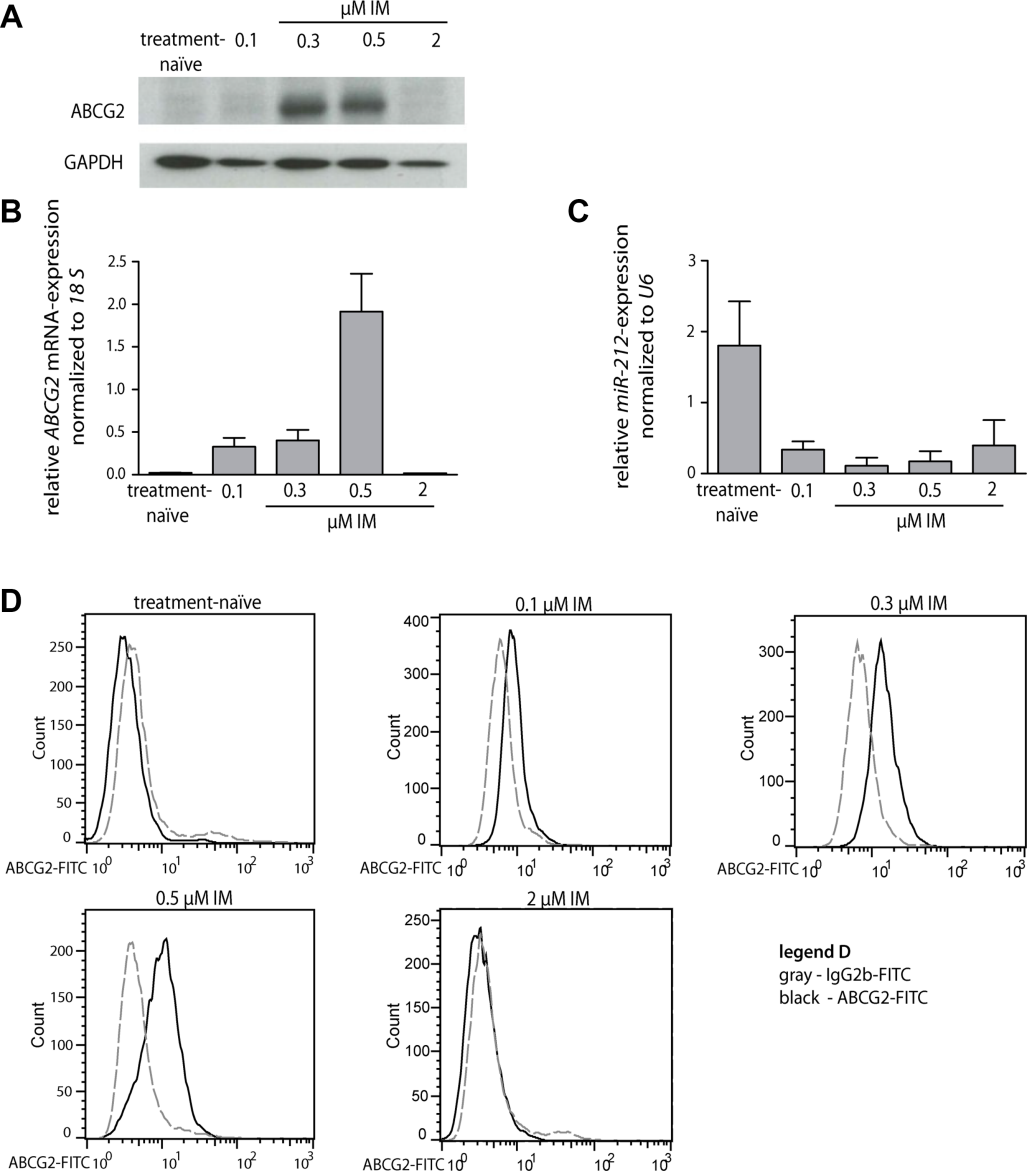
miR-212 and ABCG2 mRNA and protein expression during the development of imatinib-resistance in K-562 cells (**A**) Western blot analysis of ABCG2 protein expression during the development of IM-resistance (0.1, 0.3, 0.5 and 2 μM IM-resistant) compared to GAPDH protein expression. (**B**) *ABCG2* mRNA expression of treatment-naïve and IM-resistant cells was analyzed using qRT-PCR and normalized to *18 S*. (**C**) *miR-212* expression during the development of IM-resistant was analyzed using qRT-PCR and normalized to mammalian *U6*. (**D**) Surface staining of ABCG2 protein on treatment-naïve cells and IM-resistant sublines (0.1, 0.3, 0.5 and 2 μM IM-resistant, respectively). Gray dotted line - FITC-labeled isotype control, black solid line - FITC-conjugated ABCG2-Ab. Analyses were performed in triplicates, *n* = 3. Error bars indicate SD.

First, we analyzed the endogenous expression changes of miR-212 and ABCG2 mRNA and protein during the development of IM-resistance (treatment-naïve, 0.1, 0.3, 0.5 and 2 μM IM-resistant K-562 cells). We found that ABCG2 mRNA and protein was higher expressed at low IM doses (0.1 to 0.5 μM IM-resistant cells) compared to its expression of treatment-naïve and 2 μM IM-resistant subline (Figure 1A and 1B). Additionally, we found an increase of ABCG2 protein on the cell surface of 0.1 to 0.5 μM IM-resistant cells using flow cytometry (Figure 1D). Furthermore, we confirmed changes in miR-212 expression during the development of IM-resistance, being partly inverse correlated to ABCG2 expression [11], (Figure 1C).

Interestingly, we only observed a marginal expression of ABCB1 mRNA or protein in treatment-naïve and IM-resistant sublines (Supplementary Figure 1). Since we did not observe changes in ABCB1 but in ABCG2 expression, we assumed a higher relevance of ABCG2 in the IM-resistant cell lines tested here.

### Effect on survival and apoptosis of treatment-naïve versus imatinib-resistant cells after miR-212 transfection

**Figure 2:**
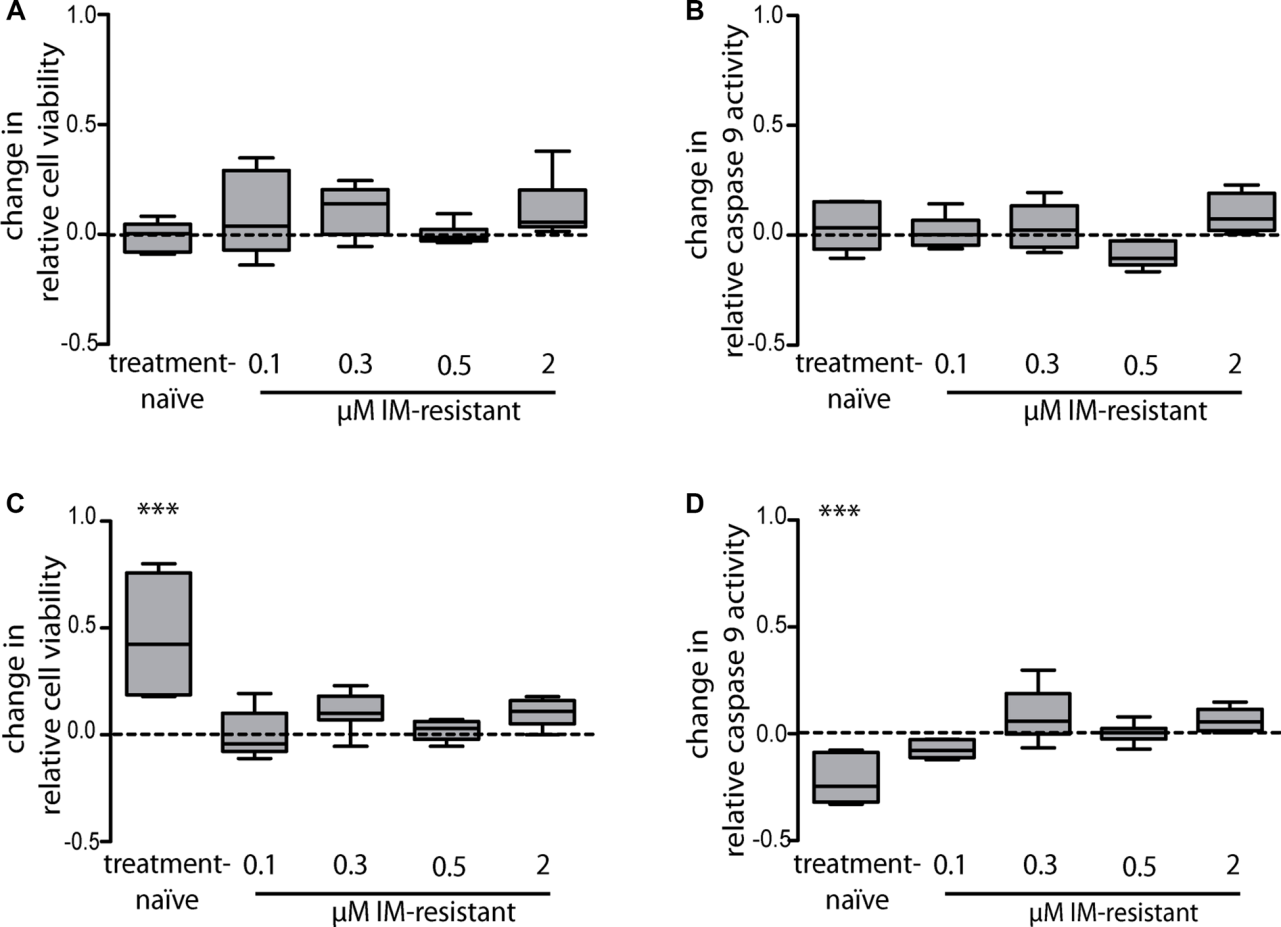
miR-212-dependent changes in imatinib-susceptibility of treatment-naïve or imatinib-resistant K-562 cells with subsequent analysis of cell viability and apoptosis Analyses of cell viability using WST-1 assay (**A**) and apoptosis using luminescent caspase 9 glo assay (**B**) after pre-miR-212 transfection and anti-miR-212 transfection (**C** and **D)**, respectively). Treatment-naïve and IM-resistant sublines were transfected with a negative control or with 25 nM miR-mimic pre-miR-212 or 75 nM miR-inhibitor anti-miR-212 and incubated with 2 μM IM for 48 h. Analyses were performed in three independent experiments. Data are normalized to respective negative control transfected cells. Statistical analysis was performed using student's *t*-test (^***^p < 0.001).

In our previous study, a direct binding of miR-212 to the *ABCG2* 3′-UTR was identified [11], which suggests that this direct regulation of ABCG2 by miR-212 might promote IM-resistance. To analyze this hypothesis, we investigated whether changes in the miR-212 expression and subsequent changes of the ABCG2 expression could be linked to altered cell survival or apoptosis under IM-treatment. Treatment-naïve and IM-resistant K-562 cells were first transfected with miR-212-mimic or -inhibitor (pre-miR/anti-miR) and then incubated with 2 μM IM for 48 h. Transfection of 25 nM miR-212-mimic and subsequent 2 μM IM-treatment did not result in any change in cell viability or apoptosis (Figure 2A and 2B). In contrast, after transfection of 75 nM anti-miR and IM-treatment, treatment-naïve cells responded with a 1.4 fold increase of cell viability in the WST-1 assay (*p* = 0.01) and a 22% decrease of apoptosis determined by the luminescent caspase 9 glo assay (*p* = 0.01) compared to negative control transfected cells (Figure 2C and 2D).

In all K-562 cells being resistant against IM-concentrations of 0.1 μM to 2 μM, neither cell viability nor apoptosis rate was changed after transfection with the miR-212-mimic or –inhibitor. These results suggest that in the presence of IM, inhibition of endogenous miR-212 promoted cell survival in IM-sensitive cells, but not in IM-resistant cells.

In our previous study, we found that another miRNA, namely miR-328 was also inversely correlated to ABCG2 expression during IM-resistance, but could not confirm any binding of this miRNA to the *ABCG2* 3′-UTR [11]. To further proof this initial observation, we also tested if transfection of miR-328-mimics or -inhibitors changed IM-susceptibility of treatment-naïve or IM-resistant cells. However, we did not observe effects on cell viability or caspase activity (Supplementary Figure 2).

### Cellular effects and changes on treatment-naïve cells after miR-212 inhibition

**Figure 3:**
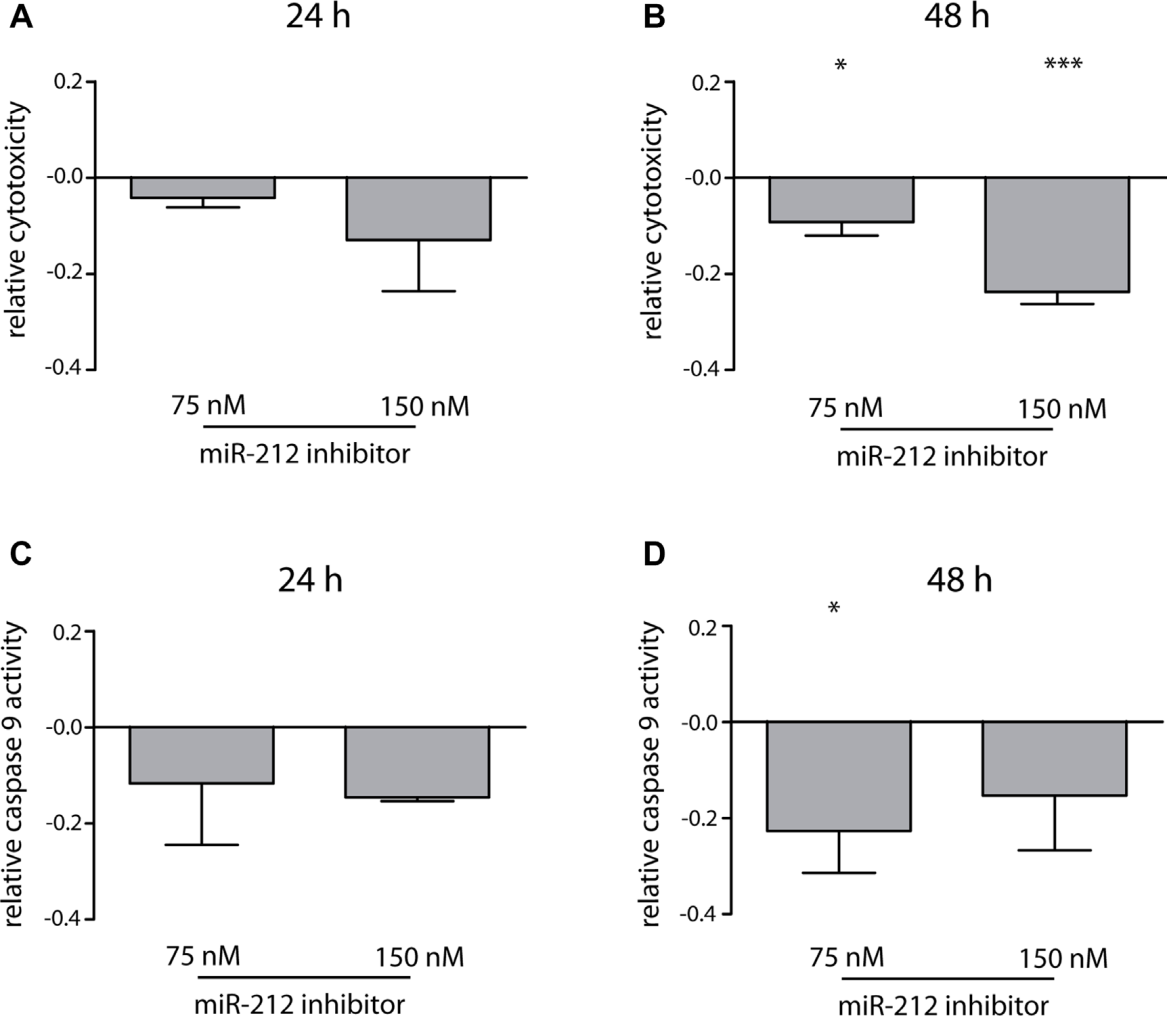
Effects of miR-212 inhibition on cell toxicity and apoptosis in treatment-naïve K-562 cells Analysis of changes in cytotoxicity 24 h (**A**) and 48 h (**B**) and caspase 9 activity 24 h (**C**) and 48 h (**D**) after miR-212 inhibition using 75 nM and 150 nM LNA power inhibitor and 2 μM IM. Analyses were performed in three independent experiments. Data normalized to negative control; error bars indicate SD, statistical analysis was performed using one-way ANOVA and Dunnet's test, (^*^p < 0.05, ^***^p < 0.001).

To test, whether the inhibitory effects on miR-212 in treatment-naïve cells were time-and concentration-dependent, we analyzed miR-212 inhibitor effects (75 and 150 nM) on cells 24 h and 48 h after transfection using LNA power inhibitors and 2 μM IM-incubation. Interestingly, 24 h after LNA-power inhibitor transfection only marginal effects on apoptosis or cytotoxicity were observed, while statistically significant effects were observed 48 h after transfection on apoptosis (*p* = 0.01, 18% decrease) and cytotoxicity (*p* = 0.03, 10% decrease) (Figure 3) confirming data of initial experiments using anti-miR-212 molecules. Doubling of inhibitor concentration led to a 24% decrease in cytotoxicity (*p* = 0.0003), but not to an escalated inhibition of apoptosis. Therefore, we continued using 75 nM inhibitor for further experiments.

### Functional analysis of miR-212 inhibition and ABCG2 upregulation on ABCG2-dependent efflux

In our earlier study, we demonstrated that miR-212 expression was inversely correlated to ABCG2 expression, being reduced at low IM-concentrations, whereas it was upregulated at higher IM-doses [11] suggesting that the effect of miR-212 inhibition on cell survival was directly linked to ABCG2 expression.

**Figure 4:**
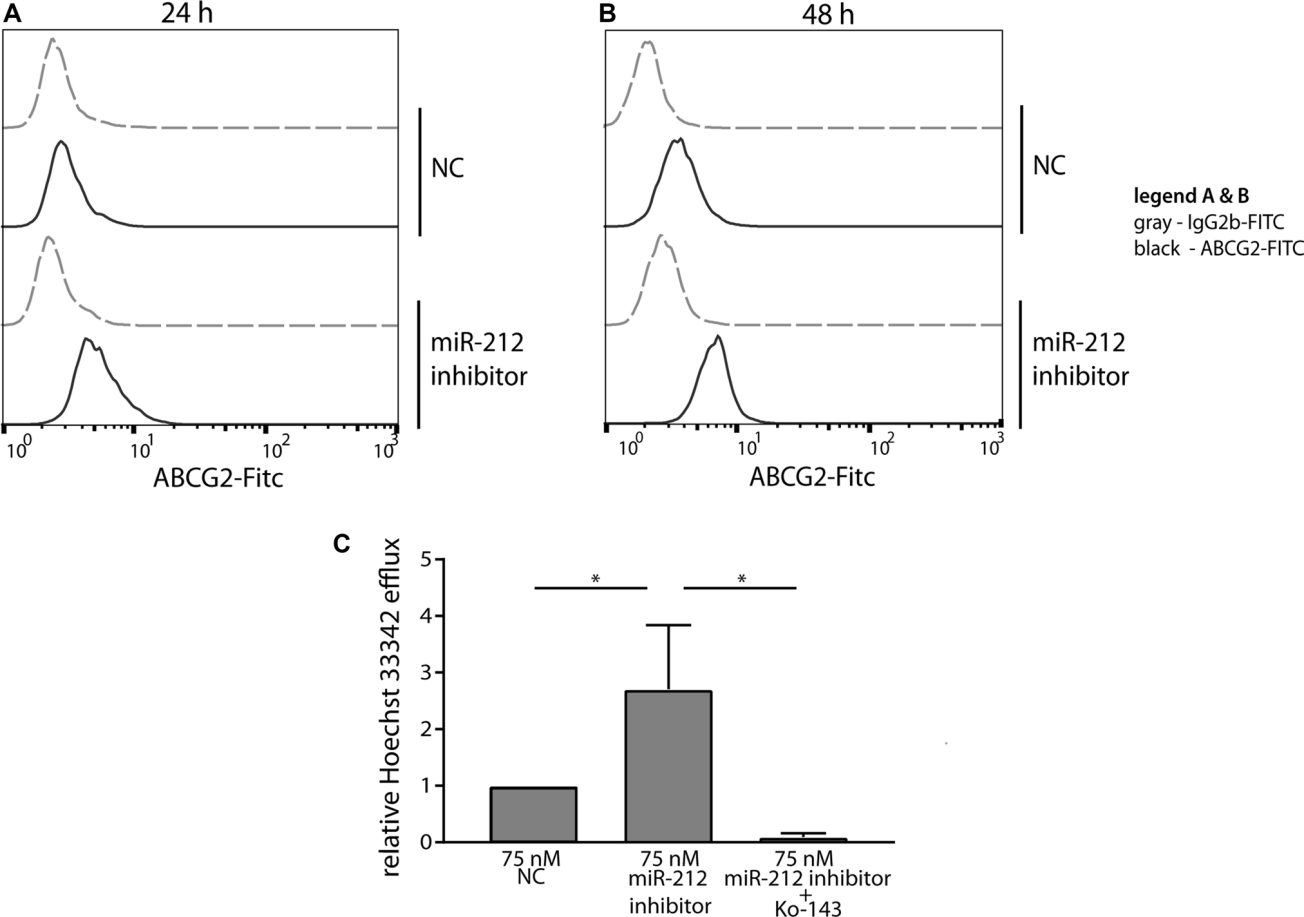
Functional analysis of miR-212 inhibition on ABCG2 protein expression and ABCG2-dependent efflux Surface staining of ABCG2 24 h (**A**) and 48 h (**B**) after transfection of treatment-naïve cells with 75 nM miR-212 LNA power inhibitor is presented as indicated. Analyses were performed in three independent experiments. Gray - FITC-labeled isotype control, black - FITC-conjugated ABCG2-Ab. (**C**) ABCG2-dependent transport was measured 24 h after transfection of treatment-naïve cells with 75 nM miR-212 power inhibitor by analyzing Hoechst 33342 fluorescence in supernatant after 30 min incubation. Data were normalized to 0 min samples of the respective sample and to the negative control transfected cells (NC). Analyses were performed in three independent experiments. Error bars indicate SD, statistical analysis was performed using one-way ANOVA and Dunnet's test (^*^p < 0.05).

In treatment-naïve cells, ABCG2 protein was more abundant on the cell surface already 24 h after miR-212 LNA power inhibitor-transfection compared to the negative control cells (Figure 4A). This observation was still seen 48 h after transfection (Figure 4B). These data indicate a direct link of miR-212 function and ABCG2 protein expression during the formation of IM-resistance. Next, we asked whether the increased ABCG2 protein expression on the cell surface leads to increased ABCG2-dependent efflux using a functional assay. Therefore, a transport assay was applied to determine the Hoechst 33342-efflux of treatment-naïve K-562 cells after transfection of 75 nM miR-212 power inhibitor (Figure 4C). Based on the upregulation of ABCG2 already 24 h after miR-212 inhibition, we used this time point for transport analysis. We determined a 2.5 fold increased Hoechst 33342-efflux after miR-212 inhibition compared to negative control transfected cells (*p* = 0.02), which is in accordance to the increase in Hoechst 33342-efflux of 0.5 μM IM-resistant cells compared to treatment-naïve cells (Supplementary Figure 3). This increased efflux was mediated by ABCG2 as the transport was abolished by the presence of the ABCG2-specific inhibitor Ko-143 (*p* = 0.03). These data confirmed that the upregulation of ABCG2 protein through miR-212 inhibition led to pronounced ABCG2-mediated transport and could therefore facilitate IM-efflux out of the cells in early IM-resistance.

### MiR-212 and ABCG2 methylation during the development of IM-resistance

To examine if differential methylation of the promoter regions of *miR-212* and *ABCG2* could explain the changes in gene expression during the development of IM- resistance, we analyzed CpGs in the promoter region of each target gene by bisulfite-pyrosequencing.

**Figure 5:**
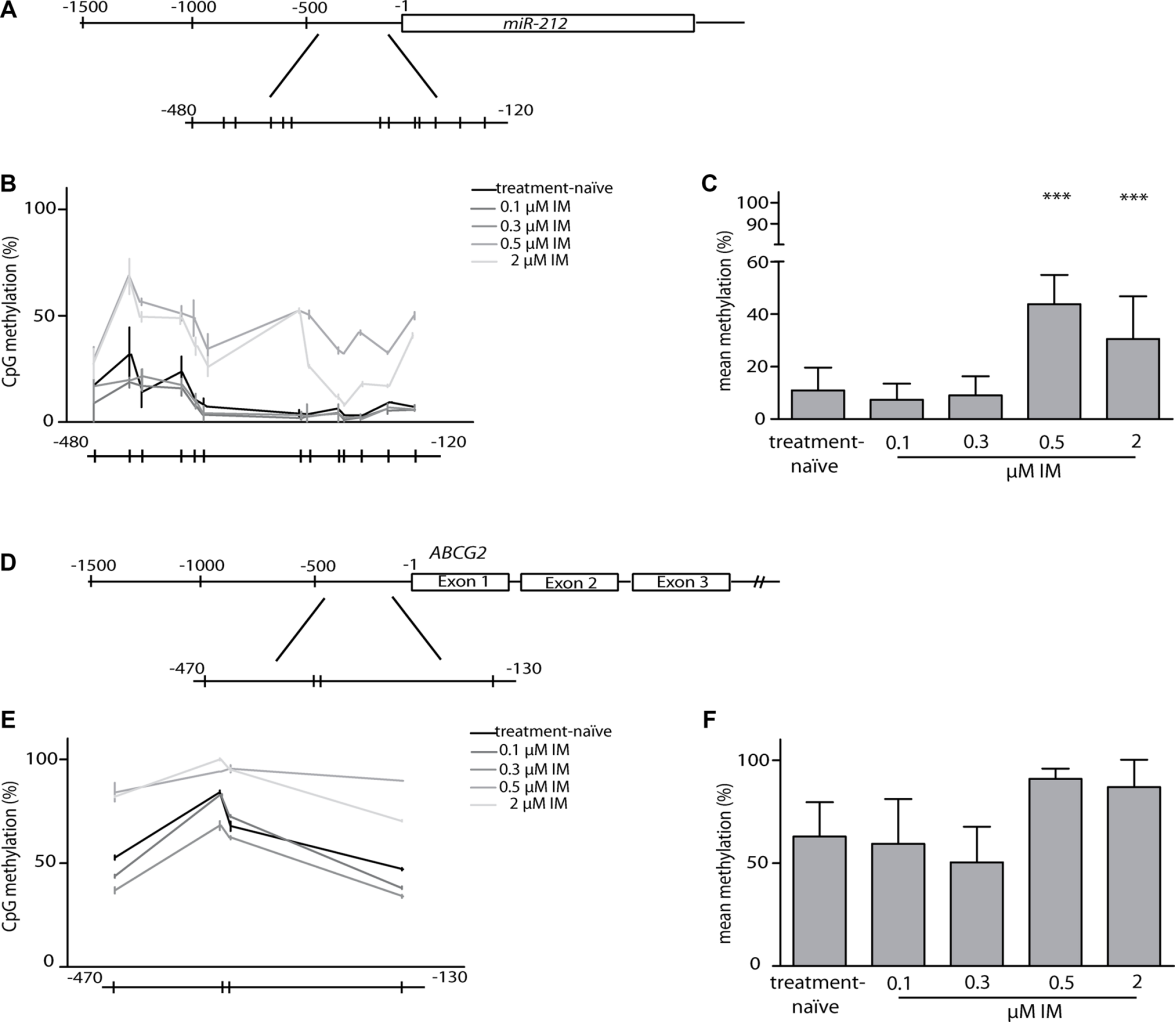
Analyses of methylation in miR-212 and ABCG2 promoter regions (**A**) Graphical overview of the *miR-212* promoter. Methylation of 13 CpGs within region –480 to –120 bp upstream of the first nucleotide of the *miR-212* gene was analyzed using bisulfite-pyrosequencing. Every line indicates one CpG. (**B**) Comparison of *miR-212* promoter methylation between treatment-naïve and IM-resistant sublines, shown as progression chart of all measured CpGs. (**C**) CpG mean methylation in treatment-naïve and IM-resistant sublines. (**D**) Graphical overview of the *ABCG2* promoter. 4 CpGs in a region between –470 and –130 bp upstream the transcription start were analyzed using bisulfite-pyrosequencing. (**E**) Comparison of *ABCG2* promoter methylation between treatment-naïve and IM-resistant sublines, shown as progression chart of all measured CpGs. (**F**) CpG mean methylation in treatment-naïve and IM-resistant sublines. Analyses were performed in three independent experiments, error bars indicate SD, statistical analysis was performed using one-way ANOVA and Dunnet's test (^***^p < 0.001).

The transcription region, as well as the promoter region of *miR-212* are located in a CpG-rich area. Therefore, we analyzed 13 CpG sites located in the CpG island of the promoter region from –480 bp to –120 bp upstream the transcription start (Figure 5A). As illustrated in Figure 5B and 5C, significant increase in DNA-hypermethylation of CpG-sites was detected in 0.5 μM (*p* = 0.0001) and 2 μM (*p* = 0.0008) IM-resistant cells. For *ABCG2*, we focused on four CpGs in the immediate proximity of the transcription start (−470 to −130 bp upstream the transcription start), (Figure 5D) as there is no CpG island located in the promoter region. Here, we did not detect any significant changes in methylation between treatment-naïve, low and high IM-resistant cells (Figure 5E and 5F). Additionally, we analyzed 18 CpG sites in the CpG island of the promoter region of *ABCB1* (−500 to −280 bp upstream the transcription start). Consistent with our results of the protein and gene expression analysis (Supplementary Figure 1), we did not observe significant alterations in methylation of the *ABCB1* promotor during the development of IM-resistance (Supplementary Figure 4).

## DISCUSSION

BCR-ABL-independent resistances are of major concern in CML therapy. In particular, dysregulation of ABC-transporters such as ABCB1 or ABCG2 or changes in microRNA-expression patterns are in the focus of research. In this study, we analyzed whether alteration of the endogenous miR-212 level led to changes in IM-susceptibility in the K-562 CML cell line. We observed that inhibition of miR-212 promoted cell viability and survival in IM-treatment-naïve cells. We showed that during the development of IM-resistance, miR-212 expression was downregulated at low IM-concentrations (0.1 to 0.5 μM IM), while ABCG2 protein was upregulated resulting in pronounced cell survival, confirming earlier studies [11]. In fact, we could mimic this mechanism by miR-212 inhibition and blockade of miR-212 function in IM-sensitive cells. We further confirmed that decrease of miR-212 led to upregulation of ABCG2 protein on the cell surface and hence, to increased ABCG2-mediated efflux. In this context, the inhibition of miR-212 followed by upregulation of ABCG2 might lead to reduction of intracellular IM-concentrations. Our data suggest that these mechanisms contribute to acute resistance against low doses of IM.

ABCG2, together with ABCB1, is one of the major efflux transporters of TKIs and is strongly expressed in drug-resistant cancer cells. However, the role of these two transporters in IM-resistance is controversially discussed. There are also controversial findings on the role of the uptake transporter organic cation transporter 1 (OCT1) [31, 32], suggesting however that for K-562 cells, OCT1 is of minor if any importance. Here, we demonstrate an upregulation of ABCG2 during IM-resistance, but no effects on ABCB1 level, while Eadie *et al*. showed only an upregulation of ABCB1 during the development of IM-resistance [7, 8]. In their experimental settings, the doses of IM were much higher and the ABCB1 upregulation was observed at these higher IM-concentrations. In contrast, the IM-concentrations we used in our experiments ranged between 0.1 and 2 μM within, which mirrors IM-plasma trough concentrations of 0.34 to 3.4 μM observed under clinical conditions in CML patients [33]. Although clinically desired maximal imatinib plasma concentrations are 2 μM, the mean concentration may be lower considering an elimination half-life of 18 hours. Moreover, due to several reasons, not all CML cells will be exposed to the full dose. Body compartments (i.e. bone marrow) are not fully accessible for imatinib, hence cells are prevented from full-dose imatinib [34]. Second, imatinib concentration varies over time, being influenced by genetic factors, esp. in cytochrome P540 3A4 (CYP3A4), concomitant diseases, i.e. liver, renal or gastrointestinal disorders, or co-administered drugs, i.e. CYP3A4 inhibitors and inducers [35–38]. Our data suggest that exposure to low concentrations of imatinib could contribute to the phenomena of imatinib-resistance, hence our data is important for the understanding of the molecular processes.

ABCG2 itself is highly expressed in stem cells and it was shown that changes in ABCG2 protein expression influence cell survival during the development of IM-resistance [4, 11, 13, 39]. Interestingly, we found that ABCG2 was upregulated only in cells resistant to low IM doses up to 0.5 μM, but not in cells resistant to 2 μM IM. This suggests an impact of drug transporters, especially ABCG2, at the early stages of IM-resistance, at low IM doses. Our findings are in line with the concept of a variable, dynamic expression of drug transporters during the development of resistance [12].

At higher IM doses, beyond 0.5 μM respectively, the mechanisms of resistance seem to be independent from ABCG2. It seems likely that other mechanisms of resistance such as upregulation of other transporters [40–42] or drifts to alternate pathways take place [43].

Furthermore, we investigated if methylation was affected during the development of IM-resistance. It is widely known that global changes in methylation occur in cancer, often leading to hypermethylation on tumor suppressor genes and hypomethylation of oncogenes [44]. Heller *et al.* showed that overall methylation status was increased in progressed CML stages compared to early CML stages of patients [45]. It was demonstrated that IM-resistant CML patients displayed an overall increase of DNA methylation, compared to IM-responsive patients [46]. To this end, we were interested whether DNA methylation of *ABCB1*, *ABCG2* and *miR-212* was altered in our *in vitro*-resistance model. We observed a hypermethylation of *miR-212* promoter as well as an elevated miR-212 expression in high dose-IM-resistant cells, compared to 0.1 and 0.3 μM-IM-resistant cells. Nevertheless, it was shown that *miR-212* promoter hypermethylation resulted in low miR-212 expression and pronounced tumor metastasis and poor prognosis in gastric cancer [30, 47]. Promoter analysis using the ENCODE database on USCS genome browser and PROMO 3.0 revealed that the examined CpGs lay within a region with binding sites for transcriptional repressors, such as RE1-silencing transcription factor (REST) and CCCTC-binding factor (CTCF). So, we suppose that hypermethylation of the examined CpGs results in less binding of these transcriptional repressors and subsequently, pronounced transcription of *miR-212* [48, 49]. Still, it is not clear if the observed hypermethylation is a cause or consequence of IM-treatment. Further experiments could now reveal how *miR-212* hypermethylation is regulated and might contribute to IM-resistance. Nevertheless, our results point to a potential epigenetic regulation of miR-212 expression during the development of IM-resistance.

To analyze *ABCG2* methylation, we examined 4 CpGs in a region of 360 bp in the *ABCG2* promoter region. We did not observe any changes in methylation of these 4 CpGs during the development of IM-resistance, these findings are in line with those of different cancer entities, such as glioblastoma multiforme [50], breast-cancer [26] or multiple myeloma [51], where no or only partial impact of *ABCG2* methylation could be observed. For *ABCB1*, we did not observe changes in methylation during the development of IM-resistance. This is in line with the absence of ABCB1 in the tested cell lines. Our findings indicate neither a regulation of *ABCG2* nor *ABCB1* by methylation in IM-resistance.

In our *in vitro*-approach, we showed that the miR-212/ABCG2-axis directly influenced cellular IM-susceptibility in early IM-resistance. Nevertheless, it is well known that a single microRNA is able to bind and regulate multiple targets in parallel [52]. MiR-212 expression is altered in a variety of tumors such as non-small cell lung cancer or hepatocellular carcinoma. In non-small cell lung cancer, miR-212 increases TRAIL-mediated cell death by targeting anti-apoptotic protein PED/PEA-15 (phosphoprotein enriched in astrocytes 15) [21] and targets synaptic acetylcholinesterase functioning as a tumor suppressor [53]. In hematologic malignancies, especially chronic lymphocytic leukemia, miR-212 is dysregulated in B cell development and proliferation, being upregulated by B cell receptor stimulation. Dysregulated targets in this process are stem cell factor SOX4 and MYC [20, 54, 55]. SOX4 also promotes progression in BCR-ABL positive acute lymphoblastic leukemia [56]. Consequently, analysis of other pathways and miR-212 targets could reveal further mechanisms causing the observed miR-212 effect on IM-susceptibility, especially in cells that underwent long-term or high dose treatment with IM.

We suppose a direct contribution of the miR-212/ABCG2-axis to cell survival in treatment-naïve, IM-sensitive cells, being decoupled in IM-resistant cells. As the ABCG2 expression in treatment-naïve cells is already very low, additional reduction of this expression by miR-212 mimics and promotion of cell death was not possible. We tested several pre-miR-concentrations, lying within the non-toxic range with lowest off target-effect [57], but did not observe any effects after transfection. In IM-resistant cells, however, additional transfection of miR-212-mimic or -inhibitor did not result in significant changes of cell viability or apoptosis as well. In these cells, other mechanisms could protect the cell from miR-212-mediated effects on ABCG2 expression, such as intracellular depletion of miR-212 or activation of counteracting pathways. Interestingly, the effects of miR-212 inhibition on apoptosis and cytotoxicity could not be significantly increased by dose doubling of the inhibitor. Potentially, the endogenous amount of miR-212 was already blocked by the lower dose of miR-212 inhibitor. Therefore, we used the lower inhibitor concentration for further experiments.

Another explanation for the decoupling of the miR-212/ABCG2-axis during development of IM-resistance could be that ABCG2 might escape miR-212 regulation by expression of shortened 3′-UTRs. To *et al*. demonstrated in a colon cancer cell line, that drug-resistant cells escaped miR-519c regulation by expression of alternate 3′-UTR lengths lacking the validated miR-519c-seed region [14]. Therefore, further emphasis should be taken on the analysis of mechanisms leading to alternate 3′-UTR length expression that might contribute to long-term IM-resistance. Another mechanism, which could contribute to decoupling of the miR-212/ABCG2-axis might be the presence of single nucleotide polymorphisms. It is well known that the variable activity or expression of drug transporters leads to differences in drug efflux and turnover [58, 59]. For ABCG2, the most important SNPs are 34G > A, 376C > T and 421C > A to affect transport of i.e. statins and potentially tyrosine kinase inhibitors such as imatinib [58]. Regarding 421C > A, Ripperger *et al*. showed that the corresponding amino acid-exchange from glutamine to lysine did not only influence protein stability promoting degradation, but also increased microRNA-dependent ABCG2 downregulation via interaction with the *ABCG2* 3′-UTR [60]. Further analyses on SNP variants in our IM-resistant cells might help understanding if ABCG2 expression or activity is altered due to SNP in exons influencing microRNA-function or if SNPs in the *ABCG2* 3′-UTR inhibit binding of miR-212 and by this contribute to miR-212-dependent ABCG2-downregulation.

Overall, our data suggest a direct interaction of miR-212 and ABCG2, promoting cell survival against IM in treatment-naïve K-562 cells. These findings could reveal new insights into the initiation of IM-resistance.

## MATERIALS AND METHODS

### Chemicals and reagents

If not indicated otherwise, chemicals were purchased from Sigma-Aldrich (Munich, Germany) or Carl Roth (Karlsruhe, Germany).

### Cell culture

K-562 cells obtained from the German Collection of Microorganisms and Cell Cultures (DSMZ, Braunschweig, Germany) were maintained as previously described [11].

### Generation of imatinib-resistant cells

IM was a kind gift of Novartis (Basel, Switzerland). It was stored at –20°C in 10 mM aqueous stock solutions and diluted to 100 μM working solutions in RPMI 1640 (Gibco, Darmstadt, Germany). IM-resistant cells were generated as previously described [11, 61, 62]. Briefly, K-562 cells were grown with increasing concentrations of IM up to a final concentration of 2 μM over a period of 3 months. Cells were considered to be resistant, when the proliferation rate under the respective IM treatment was restored. During the course of the IM-resistance, cells (resistant to distinct concentrations, referred to as K-562 cells 0.1 μM, 0.3 μM, 0.5 μM and 2 μM) were stored at –80°C until further use. The identity of all cell lines, treatment-naïve and IM-resistant, have been validated by STR-profiling using the Stem Elite ID Kit (Promega, Mannheim, Germany).

### Analysis of *BCR-ABL* mutations

Mutations of the BCR-ABL kinase domain were analyzed as previously described [63, 64]. cDNA of treatment-naïve and IM-resistant cells was obtained as described above and transferred to a nested PCR setting. Amplified PCR products were purified using the GeneJet Gel Extraction Kit (Life Technologies/Thermo Scientific) according to the manufacturer's recommendations. Sequencing reaction was performed using the previously described primers at the institute of clinical molecular biology, UKSH, Kiel [63, 64]. Sequencing results were analyzed for mutations using the BioEdit Software (Tom Hall, Ibis Bioscience, Carlsbad, California, USA). None of the cell lines showed mutations in *BCR-ABL*.

### Transfection of K-562 cells

K-562 cells were transfected by nucleofection using the Amaxa Nucleofector Kit V and Nucleofector 2b device (Lonza, Cologne, Germany) according to the manufacturer's protocol for K-562 cells. 2 × 10^6^ cells were used for each transfection and incubated in 2 ml RPMI with 10% v/v fetal calf serum (FCS, Biochrom, Berlin, Germany) and 1% v/v penicillin/streptomycin (Gibco) at 37°C for 6 h after transfection. Precursor microRNA negative control (AM17110), microRNA-mimic pre-miR-212-3p (PM10340) and pre-miR-328-3p (PM10034) (Life Technologies, Darmstadt, Germany), were transfected using a concentration of 25 nM. For microRNA inhibition, anti-miR-negative control (AM17010), anti-miR-212-3p (AM10340) and anti-miR-328-3p (AM10034) (Life Technologies) 75 nM were used. Locked Nucleic Acid (LNA) power inhibitors (negative control A (206065) and hsa-miR-212-3p (130783)) were obtained from Exiqon (Vedbaek, Denmark) and were used at concentrations of 75 nM and 150 nM. The used concentrations were proven as non-toxic for the cells.

### Analysis of effects on cell viability, apoptosis and cytotoxicity of pre-miR- or anti-miR-transfection on K-562 cells

6 h after transfection, cells were subjected to various experiments to analyze the microRNA mimic or microRNA inhibitor effects on cell viability, apoptosis and survival. For WST-1 assay, 5 × 10^4^ cells/100 μl cell culture medium and for all other assays, 5 × 10^4^ cells/50 μl medium were seeded in 96 well-plates and exposed to 2 μM IM for different time periods (24 h, 48 h) at 37°C and 5% CO_2_. After incubation, multiple assays were performed in parallel. The WST-1 assay (Roche) was used to analyze effects on the respiratory chain function to measure cell viability according to the manufacturer's recommendation. In brief, a 1:10 dilution of WST-1 reagent was added to the wells, the plate was incubated at 37°C for 2 h and detection was performed at the Infinite M200 pro device (Tecan, Crailsheim, Germany) at 440 nm and 600 nm. The luminescence-based Caspase Glo 9 Assay (Promega) was used to investigate potential effects on apoptosis. Briefly, a 1:1 ratio of Caspase Glo reagent (containing the substrate and MG-132 inhibitor) was added and plate incubation was performed according to the manufacturer's protocol. For analysis of cell survival, in terms of dead-cell protease activity released from membrane-disrupted cells, CytoTox-Glo Cytotoxicity Assay (Promega) was performed by using a 1:1 ratio of CytoTox Glo reagent and cell suspension according to the manufacturer's instruction. Detection of luminescence of both assays was performed on the Veritas microplate luminometer device and the Veritas software (Turner Biosystems/Promega). All assays were analyzed normalizing IM-treated to non-treated cells with subsequent statistical analyses as described in the statistic section.

### Analysis of mRNA and miRNA-expression

Total RNA of cells was isolated using the RNeasy Mini Kit (Qiagen, Hilden, Germany) according to the manufacturer's recommendations. For mRNA analyses, 150 ng total RNA was reversely transcribed using random hexamer primers and the Transcriptor High Fidelity cDNA Synthesis Kit (Roche, Mannheim, Germany) on the GeneAmp PCR System 9700 device (Applied Biosystems, Carlsbad, California, USA) according to the manufacturer's protocol. For miRNA analysis, RNA was reversely transcribed using microRNA Reverse Transcription Kit (Applied Biosystems) using RT primers for has-miR-212-3p (000515) and U6 snRNA (001973) according to the manufacturer's recommendations. qRT-PCR of *ABCG2* mRNA (Hs01053790_m1) and *ABCB1* mRNA (Hs00184500_m1) was performed in triplicates using stable expressed *18S* (Hs99999901_s1), for hsa-miR-212-3p using U6 snRNA as internal control, with Universal Master Mix II, without UNG (Applied Biosystems) on the ABI Prism 7900 HT device (Applied Biosystems) under default cycling conditions.

### Whole cell extracts

Whole cell lysates were generated using 1 × 10^6^ cells for each sample. The cells were pelleted at 200 × g and the supernatant was discarded. The pellets were washed once with ice-cold PBS and resuspended in denaturing lysis buffer [20 mM Tris, pH 7.4; 2% w/v sodium dodecyl sulfate (SDS); 1% v/v protease inhibitor], incubated at 95°C for 5 min, briefly sonicated and centrifuged at 15,000 × g for 15 min, 4°C to remove insoluble material. Protein extracts were stored at –80°C.

### Western blot

20 μg of protein extract were separated on 10 or 12% v/v SDS-polyacrylamide gels and transferred to polyvinylidene difluoride transfer membranes (Millipore, Schwalbach, Germany). The membranes were blocked with 4% w/v non-fat dry milk in Tris-buffered saline with Tween-20 (TBST; 0.1 M Tris, pH 7.5; 0.15 M sodium chloride; 0.1% v/v Tween 20) and incubated with the primary antibodies (ABCB1: clone C219, Enzo Life Sciences, Lörrach, Germany; ABCG2: clone BXP-21, Abcam, Cambridge, UK; both diluted 1:1000 in 1% w/v blocking solution [as described above]) on a shaker overnight at 4°C. After three washing steps with TBST, the membranes were incubated with the corresponding horseradish peroxidase-conjugated secondary antibody for 30 min (mouse IgG NXA931, 1:2000 in 2% w/v blocking solution, GE Healthcare, Munich, Germany). All Western blots were developed using the enhanced chemiluminescence (ECL) system and Hyperfilm ECL (GE Healthcare). Before the staining of the housekeeper GAPDH (Clone GT239, 1:2000 in 2% w/v blocking solution, GeneTex, Irivine, USA), blots were stripped in 2% w/v SDS, 62.5 mM Tris and 100 mM 2-mercaptoethanol for 30 min at 50°C, washed with TBST and blocked and incubated with primary and secondary antibody as described above.

### ABCG2 surface staining by flow cytometry

1 × 10^5^ cells (treatment-naïve, IM-resistant or transfected cells) were used for each staining, pelleted at 200 × g for 4 min at 4°C and were washed once with cold PBS. Thereafter, cells were transferred onto a v-bottom 96 well-plate and washed once with wash buffer (PBS, containing 1% w/v bovine serum albumin [BSA]). The cells were stained with either FITC-conjugated anti-human CD338 (ABCG2) monoclonal antibody (clone 5D3) or the respective isotype control (clone MG2b-57, both BioLegend, San Diego, California, USA) for 25 min at 4°C. Following incubation, cells were washed twice with wash buffer and fixed with 1% w/v PFA/PBS and analyzed by using the FACSCalibur flow cytometer and CellQuest Pro software (BD Biosciences, Heidelberg, Germany). For further analysis, FlowJo v.10 (FlowJo LLC, Ashland, Oregon, USA) was used. As positive control, K-562 cells resistant to 0.5 μM IM were used due to their high expression of ABCG2 on the cell surface.

### ABCG2-efflux assay

To investigate changes in ABCG2-mediated efflux, a transport assay was performed using the ABCG2-substrate Hoechst 33342. 5 × 10^5^ cells (treatment-naïve or resistant sublines; negative control or LNA miR-212 inhibitor-transfected 24 h after transfection) were used for each sample. The cells were pelleted at 200 × g for 5 min at 4°C and resuspended in 1 mL RPMI 1640 without additives. The transfected cells were pre-incubated with or without the ABCG2-inhibitor Ko-143 (600 nM, Santa Cruz, Dallas, Texas, USA) for 30 min at 37°C. For all sublines, 0.5 μg/ml Hoechst 33342 (Sigma-Aldrich) was added to the samples and incubated for 30 min at 37°C. The following steps were performed at 4°C. Cells were pelleted at 200 × g for 5 min, washed with cold Opti-MEM (Life Technologies) and transferred into 200 μl cold Opti-MEM. After incubation in a water bath at 37°C for 30 min, samples were placed on ice and cells were centrifuged at 200 × g for 5 min at 4°C. The supernatants were transferred into black, clear bottom 96 well-plates and detection was performed at the Infinite M200 pro device (Tecan) at 350 nm for excitation and 460 nm for emission. Data was normalized to treatment-naïve cells or the negative control and 0 min sample of the respective transfected cells.

### Analysis of methylation

Methylation analysis of treatment-naïve and IM-resistant cells was performed using bisulfite-pyrosequencing. For this, genomic DNA was isolated using Gentra Puregene kit (Qiagen) according to the manufacturer's recommendations. 1 μg DNA was bisulfite converted using Epitect Bisulfite kit (Qiagen) according to the manufacturer's protocol. Targeted promoter specific PCR (*miR-212*, *ABCG2*, *ABCB1*) was performed as a nested PCR design using Invitrogen Platinum Taq DNA Polymerase (Thermo Scientific) following the instructions of the manufacturer, including 1% v/v DMSO for *ABCG2* PCR reactions. Primers (see Supplementary Table 1) were designed using Pyromark Assay Design Software.

Sequencing was performed by pyrosequencing using PSQ HS96 MD device (Qiagen), as described elsewhere [65] using 5 μl PCR product, no-template controls, human methylated, converted control DNA (EpiTect Control DNA, methylated, Qiagen) and unmethylated control DNA, kindly provided by the Institute of Human Genetics, Kiel.

### Software and statistics

DNA sequences (hsa-miR-212: NR_029625; ABCG2: NM_001257386, ABCB1: NM_000927) were obtained from UCSC genome browser, GRCh37/hg19 (genome.ucsc.edu). Analysis of transcription factor binding was performed using the ENCODE project database [66] on UCSC genome browser and Promo3.0 (alggen.Isi.upc.es). Unless not otherwise described, statistical analysis was performed using one-way ANOVA, Dunnet's test and/or student's *t*-test and the GraphPad software (Version 7.0 for Windows, San Diego California, USA).

## SUPPLEMENTARY MATERIALS FIGURES AND TABLE



## Abbreviations

IM: imatinib
BCR: breakpoint cluster region
ABL: Abelson tyrosine kinase
TKI: tyrosine kinase inhibitor
CML: chronic myelogenous leukemia
ABCG2/BCRP: breast cancer related protein
ABCB1/P-gp: P-glycoprotein
