# Supplementary material for: MicroRNA-212/ABCG2-axis contributes to development of imatinib-resistance in leukemic cells
Source: Oncotarget. 2017 Sep 26;8(54):92018–31. doi: 10.18632/oncotarget.21272 (PMC5696160; doi:10.18632/oncotarget.21272)
Supplement: Supplementary file 1 [file oncotarget-08-92018-s001.pdf]

MicroRNA-212/ABCG2-axis contributes to development of imatinib-resistance in leukemic cells

SUPPLEMENTARY MATERIALS

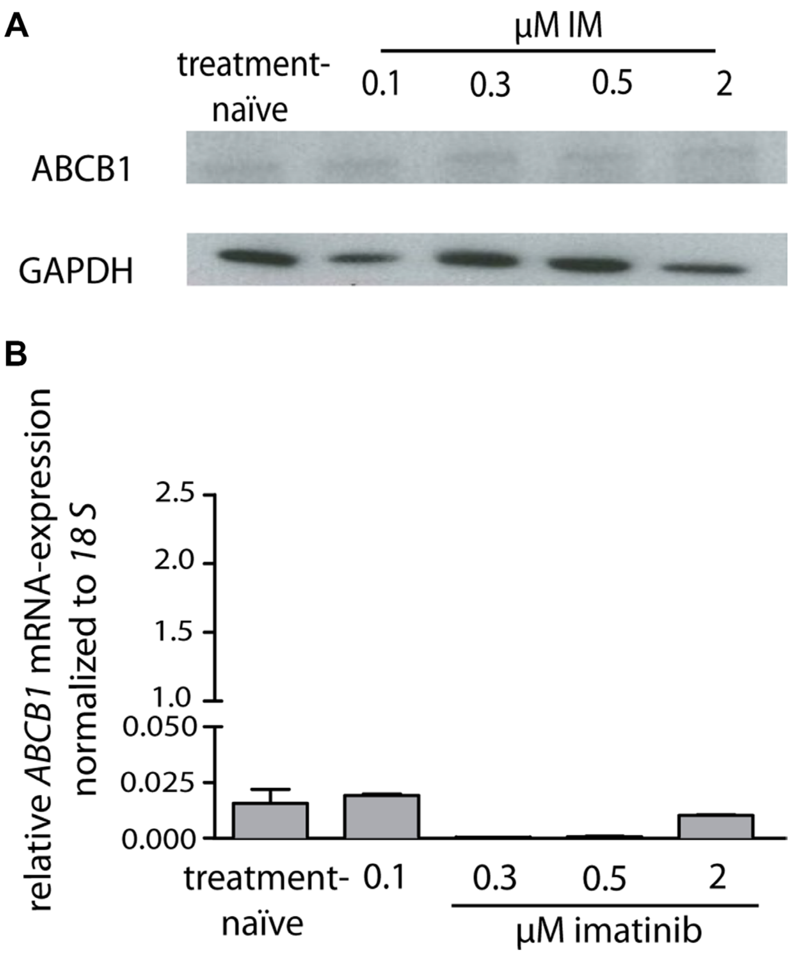

Supplementary Figure 1: ABCB1 mRNA and protein expression during the development of imatinib-resistance in K-562 cells. (**A**) Western blot analysis of ABCB1 protein expression during the development of IM-resistance (0.1, 0.3, 0.5 and 2 µM IM-resistant) compared to protein expression of GAPDH. (**B**) *ABCB1* mRNA expression of treatment-naïve and IM-resistant cells was analyzed using qRT-PCR. The data was normalized to *18 S*. Analyses were performed in triplicates, *n* = 3. Error bars indicate SD.

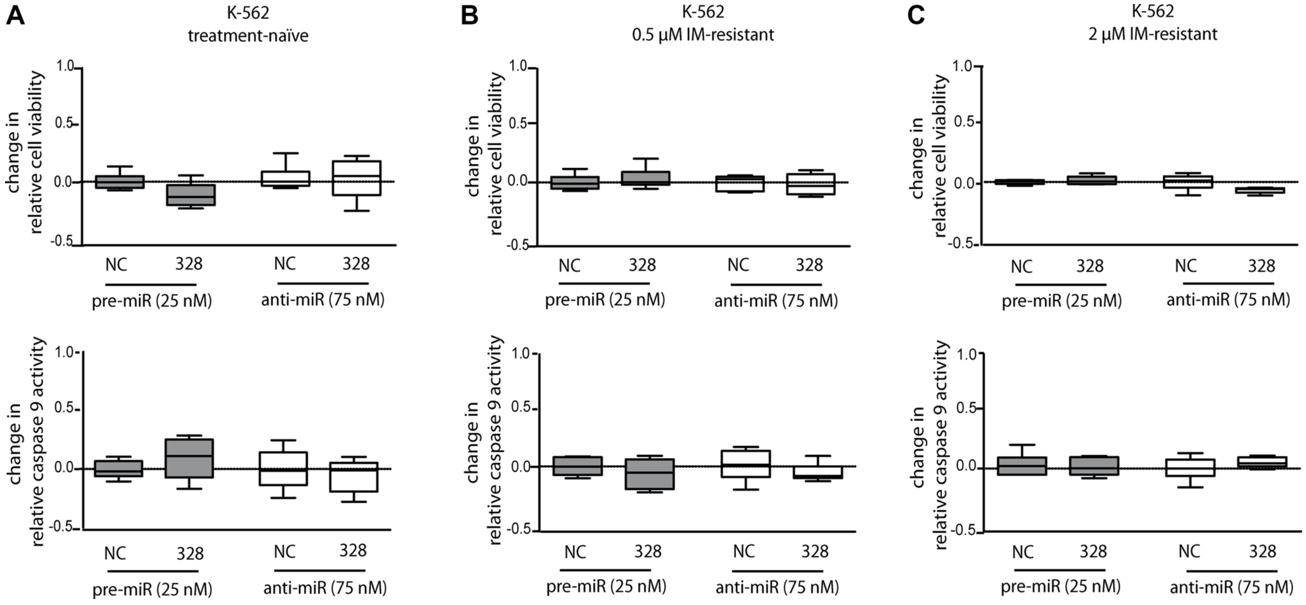

Supplementary Figure 2: miR-328-dependent changes in imatinib-susceptibility of treatment-naive or imatinib-resistant K-562 cells with subsequent analysis of cell viability and apoptosis. K-562 cells, treatment-naïve (**A**) and two resistant sublines (0.5 (**B**), 2 µM (**C**) IM) were transfected with 25 nM miR-mimic pre-miR-328 or 75 nM miR-inhibitor anti-miR-328 and incubated with 2 µM imatinib for 48 h. Analyses of cell viability using WST-1 assay (upper panels) and apoptosis using luminescent caspase 9 glo assay (lower panels) after pre-miR-212 transfection and anti-miR-212 transfection are represented normalized to negative control transfected cells. Analyses were performed in three independent experiments. Data are normalized to respective negative control transfected cells. Error bars indicate SD, statistical analysis was performed using student's *t*-test

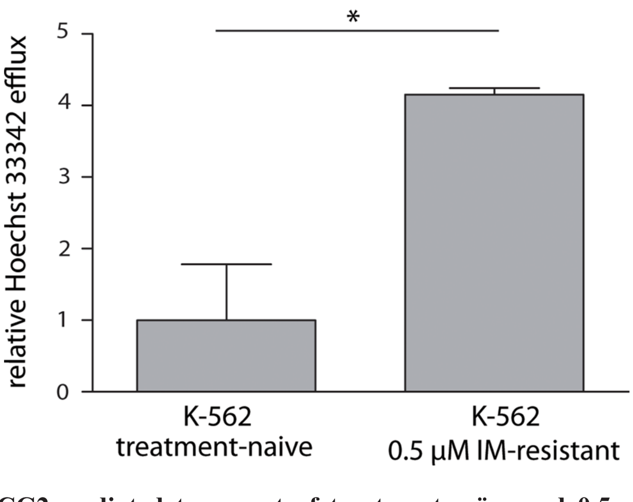

Supplementary Figure 3: ABCG2-mediated transport of treatment-naïve and 0.5 µM IM-resistance K-562 cells. ABCG2-dependent transport of treatment-naïve and 0.5 µM IM-resistant cells was investigated by analyzing Hoechst 33342 fluorescence in supernatant after 30 min incubation. Data were normalized to treatment-naïve cells. Analyses were performed in three independent experiments. Error bars indicate SD, statistical analysis was performed using student's *t*-test (*$p < 0.05$).

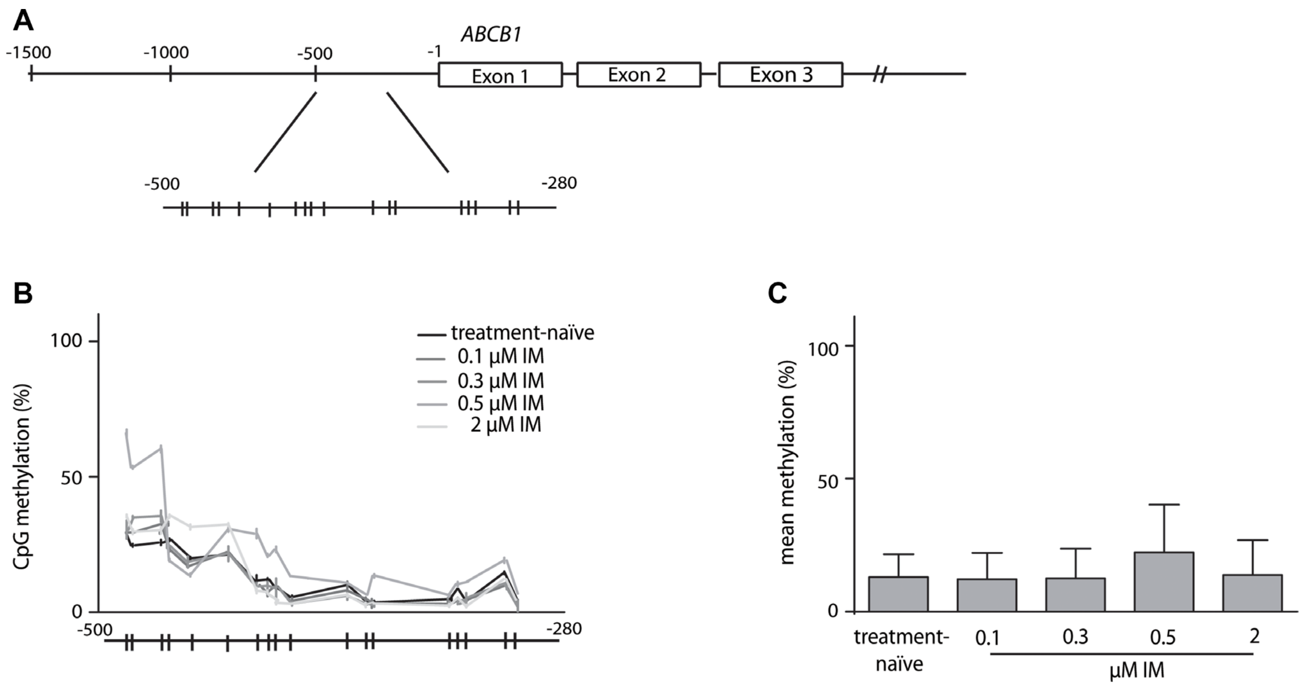

Supplementary Figure 4: Analyses of methylation in *ABCB1* promoter. (**A**) Graphical overview of the ABCB1 promoter. Methylation of 18 CpGs in a region between –500 and –280 bp upstream the transcription start were analyzed using bisulfite-sequencing. Every line indicates one CpG. (**B**) Comparison of *ABCB1* promoter methylation between treatment-naïve and IM-resistant sublines, shown as progression chart of all measured CpGs. (**C**) CpG mean methylation in treatment-naïve and IM-resistant sublines. Analyses were performed in three independent experiments; error bars indicate SD, statistical analysis was performed using one-way ANOVA and Dunnet's test.

Supplementary Table 1: Primer used for bisulfite sequencing

Primer name	Primer-sequence (5′→ 3′)	No. and position of CpGs analyzed	Annealing temperature
ABCG2_forward	TGGGATTTAAATAAAAGATTTAATGGT		50°
ABCG2_reverse	ACCTTTACATTAAAACCTAACTCTTTAT		
ABCG2_forward_nested	AATAAAAGATTTAATGGTTTTAGTT		48°
ABCG2_reverse_nested	ACATTAAAACCTAACTCTTTATT		
ABCG2_sequencing1	AAATAAAAGATTTAATGGTTTTAG	1 (chr4:89152554)	
ABCG2_sequencing2	AGGGGGAAAGTTATATG	2 (chr4:89152647-89152649)	
ABCG2_sequencing3	GGATTATGATATAATTTGAAAAGGA	1 (chr4:89152864)	
miR212_forward	GGGGTTTTTGAGTTATTTTTTTAGGAAA		53°
miR212_reverse	ATATCCCAAAAAAAACACTT		
miR212_forward_nested	AAGTGAGGAGAAGGTGTT		52°
miR212_reverse_nested	CCCCCCATCCTAAAAAAACAACTC		
miR212_sequencing1	AAGTGAGGAGAAGGTGTT	6 (chr17:1954106-1954065)	
miR212_sequencing2	TTTTTTTAGGTAGGTT	7 (chr17:1953933- 1953868)	
ABCB1_forward	GGATAGTGTGAAGTTTTTTGGTAAG		50°
ABCB1_reverse	AACACTACAAAAACTTTCCTATAC		
ABCB1_forward_nested	TGGGGTTAGATTTAGATTTAGGAGT		56°
ABCB1_reverse_nested	CAACATCTCCACCAAAACAAAATTAAAAAT		
ABCB1_sequencing1	GTAGTGGTATTGGATTATG	6 (chr7:87229856- 87229836)	
ABCB1_sequencing2	GGGATTTGTTTTTTGAGT	7 (chr7:87229810- 87229775)	
ABCB1_sequencing3	GGAATCGGGAGGGAGA	5 (chr7:87229752- 87229723)	
